# Genetic diversity and phylogenetic relationship analyzed by microsatellite markers in eight Indonesian local duck populations

**DOI:** 10.5713/ajas.18.0055

**Published:** 2018-12-21

**Authors:** Dwi Nur Happy Hariyono, Dyah Maharani, Sunghyun Cho, Prabuddha Manjula, Dongwon Seo, Nuri Choi, Jafendi Hasoloan Purba Sidadolog, Jun-Heon Lee

**Affiliations:** 1 Department of Animal Breeding and Reproduction, Faculty of Animal Science, Universitas Gadjah Mada, Yogyakarta 55281, Indonesia; 2 Division of Animal and Dairy Science, College of Agriculture and Life Sciences, Chungnam National University, Daejeon 34134, Korea

**Keywords:** Genetic Diversity, Indonesian Local Ducks, Microsatellite Markers, Phylogenetic Relationship

## Abstract

**Objective:**

At least eight local duck breeds have been recognized and documented as national germplasm of Indonesia so far. It is necessary to genetically characterize the local duck breeds for aiding conservation and future improvement strategies. Thus, this study was carried out to assess genetic diversity and phylogenetic relationship of eight local duck populations of Indonesia using microsatellite markers.

**Methods:**

In total, 240 individuals (30 individuals each population) from Alabio (AL), Bayang (BY), Magelang (MG), Mojosari (MJ), Pegagan (PG), Pitalah (PT), Rambon (RM), and Turi (TR) duck populations were genotyped using 22 microsatellite markers.

**Results:**

The results showed a moderate level of genetic diversity among populations, with a total of 153 alleles detected over all loci and populations, ranging from 3 to 22 alleles per locus. Observed (Ho) and expected heterozygosity (He), as well as polymorphism information content over all loci and populations were 0.440, 0.566, and 0.513, respectively. Heterozygote deficiency in the overall populations (*F*_IT_ = 0.237), was partly due to the heterozygote deficiency within populations (*F*_IS_ = 0.114) and moderate level of genetic differentiation among populations (*F*_ST_ = 0.137). The most diverse population was MG (He = 0.545) and the least diverse population was AL (He = 0.368). The majority of populations were relatively in heterozygote deficiency (except AL), due to inbreeding. The genetic distances, phylogenetic trees, and principal coordinates analysis concluded that the populations can be grouped into two major clusters, resulting AL, MG, and MJ in one cluster separated from the remaining populations.

**Conclusion:**

The present study revealed a considerable genetic diversity of studied populations and thus, proper management strategies should be applied to preserve genetic diversity and prevent loss of alleles.

## INTRODUCTION

Attention and awareness to genetic conservation of locally developed livestock breeds have increased in recent years, evidenced by many studies concerning genetic diversity of the breeds. Conservation of genetic diversity plays an important role in sustaining the livestock breeds. Genetic diversity within a species similarly increases the probability of survival in a range of environments [1] and provides genetic materials for future breeding programmes as well as important materials from a scientific point of view. Reducing the genetic diversity of a species means losing not only genetic ‘wealth’, but also reducing the possibility of the species to adapt to harsh environmental conditions and disease outbreaks [2].

In Indonesia, a number of local duck breeds, namely Alabio (AL), Bayang (BY), Magelang (MG), Mojosari (MJ), Pegagan (PG), Pitalah (PT), Rambon (RM), and Turi (TR) were included as important assets by the Indonesian Ministry of Agriculture, and play an important role in a socio-economic aspect as they provide livelihood to smallholders as well as food for humans. Ducks are reared by breeders for egg production and the culled ducks used for meat production. These local ducks are known for their high fitness levels under harsh conditions and ability to survive with coarse and alternative fodder. Because of these important roles, to evaluating and monitoring the genetic diversity status and phylogenetic relationship of these local breeds is highly recommended. Comprehensive knowledge of the existing genetic variability is the first step for the conservation and utilization of domestic animal biodiversity [3,4]. Conservation of local duck breeds should rely upon several sources of information, including the degree of endangerment, adaptation to a specific environment, traits of economic importance, and cultural or historical value of the breeds [5], molecular characterization may provide as an important initial guide.

Recent advances in molecular technology allow us to assess genetic diversity of livestock breeds at DNA level. Microsatellites or simple sequence repeats are recently the most favoured molecular markers for population analysis, owing to the high variability, ease, and accuracy of assaying microsatellites [6]. They may prove particularly valuable for population discrimination and genotype identification [7] due to the high level of polymorphism compared with conventional allozyme markers [8,9]. So far, employing microsatellite markers to assess ducks genetic diversity has been established by many studies [10–13] and the reported results provided clear evidence of the usefulness of microsatellites for genetic diversity studies. Using microsatellites in our samples of Indonesian local duck populations also allow comparison with published studies of local duck breeds from other countries.

In Indonesia, the use of molecular markers for assessing genetic diversity in several local duck breeds has been previously reported, including AL using 7 microsatellites [14], BY using 2 microsatellites [15] and MG using single nucleotide polymorphism (SNP) [16]. In the current study, we attempt to use 22 microsatellite markers in determining the genetic diversity and phylogenetic relationship of eight local duck populations of Indonesia. The results may prove to be valuable for the future breeding programs and conservation of the local duck breeds.

## MATERIALS AND METHODS

### Sample collection and DNA extraction

**Figure 1:**
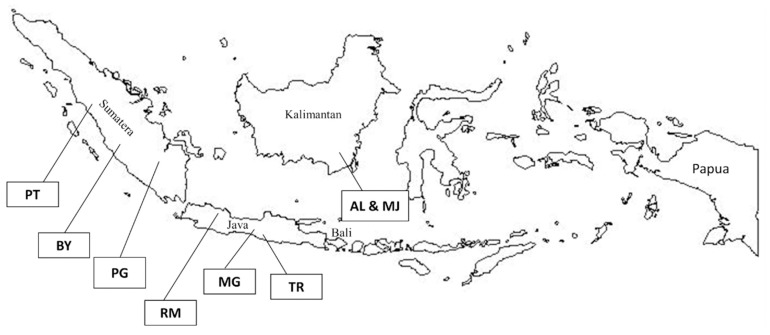
Locations of analyzed eight local duck populations in Indonesia (AL, Alabio; BY, Bayang; MG, Magelang; MJ, Mojosari; PG, Pegagan; PT, Pitalah; RM, Rambon; TR, Turi).

In total, 240 animals representing eight local duck populations in Indonesia (30 animals per population) were sampled from six provinces (Figure 1). The eight duck populations were AL and MJ from Pelaihari, South Kalimantan; BY and PT from West Sumatera, MG from Central Java; PG from South Sumatera; RM from West Java; and TR from Special Region of Yogyakarta (DIY). The blood samples were obtained from the ulnar vein using vacutainer tubes with K2-ethylenediaminetetraacetic acid anticoagulant. Genomic DNA was extracted from these blood samples using gSYNC DNA Extraction Kit (Geneaid, New Taipei City, Taiwan) following the manufacturer’s instructions and stored at −20°C before doing polymerase chain reaction (PCR) amplification. Extracted DNA samples were checked for quality and concentration by electrophoresis on 1% agarose gel, as well as by a spectrophotometer using the NanoDrop 2000C (Thermo Scientific, Waltham, MA, USA).

### Microsatellites amplification and genotyping

Twenty-two microsatellite markers distributed in 6 linkage groups and chromosomes (Table 1) were chosen based on their genomic location and their degree of polymorphism. Primer forward from each pair was modified using capillary-based dye (FAM, VIC, NED, and PET). The PCR was performed in a 20 μL volume containing 2 μL of 10 ng/μL of duck genomic DNA, 2× multi HS Prime Taq Premix (GeNet Bio, Daejeon, Korea), 8 pmol of each forward and reverse primer (Applied Biosystems, Foster City, CA, USA), and distilled water. PCR was carried out under following conditions: initial denaturation for 10 min at 95°C, followed by 38 cycles of 30 s of denaturation at 95°C, 30 s of annealing at 60°C, 30 s of extension at 72°C, and final extension for 10 min at 72°C using BIO-RAD T100 Thermal Cycler. The amplified DNA was then genotyped using Genetic Analyzer 3730xl (Applied Biosystems, USA), with genotyping reaction containing 1 μL of diluted PCR products, 10 μL of Hi-Di Formamide (Applied Biosystems, USA), and 0.1 μL of GeneScan-500 LIZ size standard marker (Applied Biosystems, USA). The GeneMapper ver.3.7 (Applied Biosystems, USA) was used for genotype identification.

### Statistical analysis

The genetic diversity among populations was determined by these indicators: number of alleles (Na), observed heterozygosity (Ho), expected heterozygosity (He), and polymorphism information content (PIC) which were estimated using Cervus ver.3.0 program [17], and *F*-statistics, including inbreeding coefficient of an individual relative to the subpopulations (*F*_IS_), inbreeding coefficient of an individual relative to the total population (*F*_IT_), and genetic differentiation index between population (*F*_ST_) which were calculated using GenAlex ver. 6.501 [18]. The software was also employed to determine genetic diversity within each population (Na, Ho, He, and *F*_IS_). For phylogenetic relationship analysis, GenAlex software was used to perform pairwise population matrices based on either *F*_ST_ or Nei’s genetic distance and to construct principal coordinates analysis (PcoA). The resulted pairwise population matrices were then used to construct phylogenetic trees using MEGA software ver. 7.0.14 [19].

## RESULTS

### Genetic diversity and differentiation analysis

Genetic diversity indicators are summarized in Table 1 and Table 2 for among and within duck populations studied, respectively. In total, 153 alleles were detected at these 22 loci in 240 individuals, with the number of alleles per locus ranging from 3 (CAUD128, AMU123, and CAUD009) to 22 (CAUD048), with an average value of 6.96 alleles per locus. Observed and expected heterozygosity values ranged from 0.026 to 0.866 and 0.079 to 0.927, respectively. The mean expected heterozygosity of 0.566 indicated medium to high levels of genetic diversity in duck populations studied. The PIC value for the loci ranged from 0.07 to 0.920, with an average of 0.513.

*F*-statistics were estimated in a fixation index as genetic differentiation (*F*_ST_), global deficit among eight duck populations (*F*_IT_), and the heterozygote deficit within duck populations (*F*_IS_), with an average value of 0.137, 0.237, and 0.114, respectively (Table 1). The average value of *F*_ST_ indicated that about 13.70% of total genetic variation corresponded to differences between populations, while 86.30% was explained by differences between individuals.

Generally, within each population, relatively low to moderate genetic diversity was observed, depicted by range values of Na, Ho, and He of 3.136 to 4.864, 0.371 to 0.488, and 0.368 to 0.545, respectively. All of the duck populations, except AL, showed a deficiency of heterozygosity, indicated by positive *F*_IS_ values, ranging from 0.037 to 0.171.

### Phylogenetic relationship analysis

**Figure 2:**
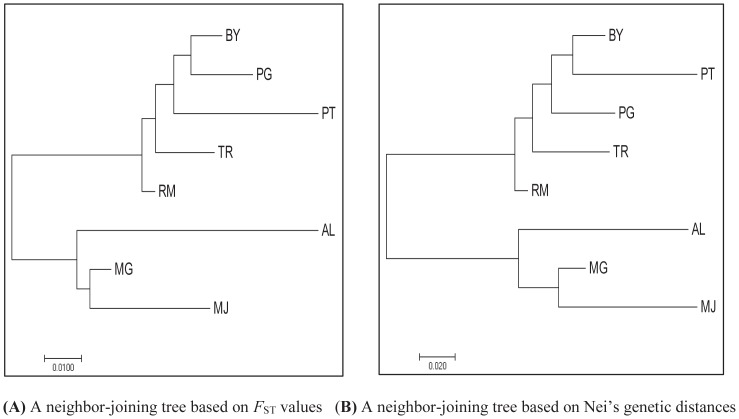
Neighbor-joining tree constructed using pairwise population matrix of *F*_ST_ values (A) and Nei’s genetic distances (B) of eight duck popullations. AL, Alabio; BY, Bayang; MG, Magelang; MJ, Mojosari; PG, Pegagan; PT, Pitalah; RM, Rambon; TR, Turi.

The pairwise *F*_ST_ value and Nei’s genetic distance across eight duck populations are shown in Table 3. The genetic distances were the shortest between RM and TR (0.021) and between BY and PG (0.051), while the least genetic relationship was between AL and PT (0.155) and between MJ and PT (0.367), based on *F*_ST_ value and Nei’s genetic distances, respectively. The matrix of *F*_ST_ value and Nei’s genetic distances was further used to construct neighbor-joining (NJ) trees (Figure 2).

**Figure 3:**
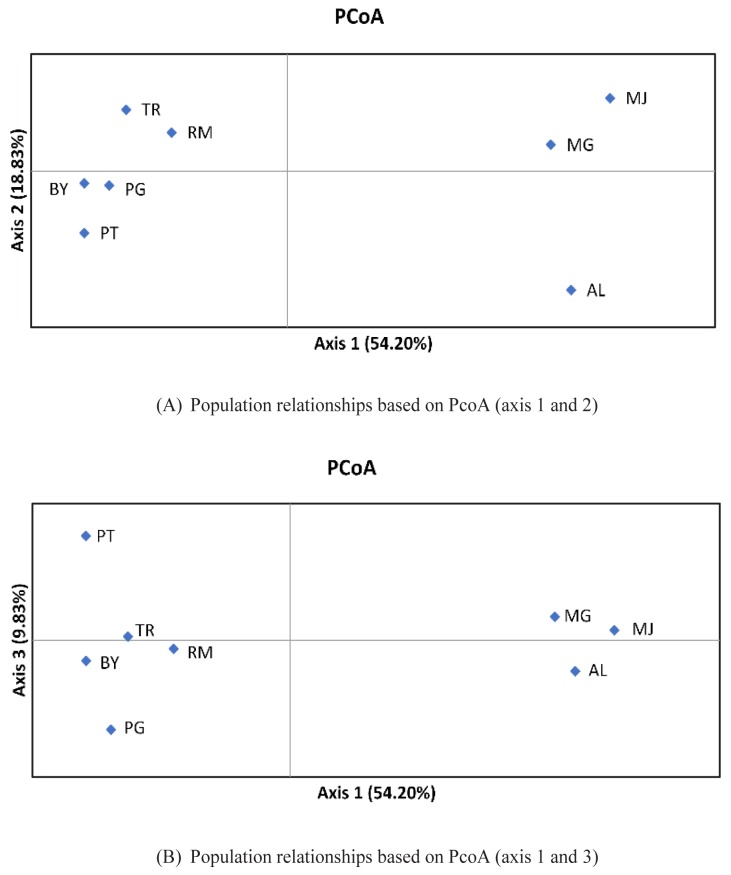
Principal coordinates anaylsis (PcoA) of based on covariance matrix of Nei’s genetic distance. (A) Population relationships based on PcoA (axis 1 and 2), (B) Population relationships based on PcoA (axis 1 and 3). AL, Alabio; BY, Bayang; MG, Magelang; MJ, Mojosari; PG, Pegagan; PT, Pitalah; RM, Rambon; TR, Turi.

The resulted NJ trees revealed relatively similar results using both matrices. Two main clusters were formed, with AL, MJ, and MG duck populations in one cluster, whereas others joined together in a different cluster. Such clustering of the duck populations into two main clusters clearly indicated that some populations originated from different provinces and or islands. A PcoA is also presented using allele frequencies of 22 loci to summarize population relationships (Figure 3). The first, second and third components accounted for 54.20%, 18.83%, and 9.83%, respectively to the total of genetic variability. AL and two populations (MG and MJ) were clearly separated into different single quadrate that differs from other populations (PcoA axis 1 and 2). In the PcoA axis 1 and 3, BY, PG, and RM formed one group that was near to TR, but still generally separated from AL, MJ, and MG populations.

## DISCUSSION

### Genetic diversity analysis

In this study, all microsatellite loci were found to be polymorphic. The average number of alleles in this study (Na = 6.96) were lower than the findings of the other studies using same microsatellite markers in Asian duck populations, with number of alleles of 9.38 [12] and 11.5 [13]. Using three same loci (CAUD011, CAUD035, and CAUD066), nine to fiveteen alleles per locus were also observed by Liu et al [10] in Chinese indigenous duck breeds. Furthermore, five alleles were detected at APH24 locus in this study. Other genetic diversity indicators, however, showed values close to zero for Ho (0.026), He (0.079), and PIC (0.077). There was no detected alleles at APH24, as reported by Ismoyowati and Purwantini [14] in Indonesian duck populations (AL and Bali). The results suggest that genetic diversity of Indonesian local ducks is lower than other Asian duck populations.

To test the informativeness of observed loci, we measured PIC, with the resulting average value of 0.513. For animal traceability, PIC>0.5 and He>0.6 are the most reasonable informative loci for application of genetics [20]. From 22 loci, twelve loci (PIC>0.5) were included as highly informative loci and were appropriate for assesing genetic diversity and population discrimination.

Based on the average value of expected heterozygosity (0.566), a moderate level of genetic diversity among populations studied was obtained. In addition, heterozygote deficiency was detected, which was depicted by the lower average value of observed compared to expected heterozygosity, as well as the positive average value of *F*_IS_. Such phenomenon can be explained by various factors such as non-random mating, unamplified alleles (“null” alleles) and subdivision in populations studied (Wahlund’s effects). The studied populations revealed a moderate genetic differentiation among eight populations (*F*_ST_ = 0.137). The results showed that genetic diversity maintained within duck populations was higher than the one preserved among duck populations. This genetic diversity could be a valuable tool for implementing future genetic improvement and conservation of duck populations in Indonesia.

The genetic diversity indicators within eight duck populations are also summarized in Table 2. The mean number of alleles observed over a range of loci in different populations is considered to be a reasonable indicator of genetic variation within the populations [21]. The mean number of alleles was lowest (3.136) in the AL duck populations, as well as the values of Ho (0.371) and He (0.368). In contrast, RM and TR duck populations were highest for the mean number of alleles while MG duck population showed highest genetic diversity compared to the others (He = 0.545). Observed heterozygosity was lower than expected heterozygosity in all populations, except AL duck population. This was also evidenced by positive values of *F*_IS_ in the seven populations, showing a departure from Hardy-Weinberg equilibrium. The disequilibrium was mainly caused by heterozygote deficiencies, as a result from the existence of inbreeding and or Wahlund effect (population substructure). Since the blood samples were collected from the same flock for each population, the existence of Wahlund effect may be ruled out. The reasonable cause of heterozygote deficiency is the presence of inbreeding. AL and MJ duck populations were reared under a intensive production system, established by livestock breeding center, while the remaining populations were reared under semi-intensive production systems by breeders in villages. Furthermore, after interviewing these breeders, it appears that often there is no pedigree record kept of the animals in semi-intensive production systems and mating between genetically related animals can occur. Unplanned and unsystematic breeding strategies may lead to the lack of sufficient number of breeding males in the breeding population. Mating between animals with similiar phenotypes also occured as the villagers believed mating between ducks with a certain feather colour produced offspring with higher egg production and thus, a panmixic population is unlikely to occur.

### Phylogenetic relationship analysis

Genetic distance indicators of *F*_ST_ values and Nei’s [22] genetic distances revealed a genetic relationship among eight duck populations. In general, relatively similar results were obtained from both indicators. Unsurprisingly, the greatest genetic distances were between MJ and PT and between AL and PT duck populations, based either *F*_ST_ values or Nei’s genetic distance. PT duck population was sampled from a highland-isolated area, while the two AL and MJ duck populations were sampled from same livestock breeding center (BPTU-HPT Pelaihari) with a controlled breeding system. Projection of population relationships by constructing NJ trees based on the two indicators clearly separated the analyzed populations into two clusters. The clustering pattern was further supported by PcoA analysis. MG and MJ duck populations were consistently grouped in one cluster that was closer to the AL duck population compared to the remaining populations. One interesting result was that RM and TR duck populations (originated from Java island) also tended to join together and clustered with BY, PG, and PT duck populations (originated from Sumatera island) in both NJ trees. In the past, many migrants from Java island moved to Sumatera island and other larger islands in Indonesia to get more land for agriculture. Therefore, it is possible for BY, PG, PT, RM, and TR duck populations to share common ancestors when the migrants brought the ducks with them to the new region. The study highlighted that geograhic distance is not always a predictor of phylogenetic relationship. However, to get better understanding of phylogenetic among populations, it is important to combine information on the basis of phenotypic and molecular genetic characterization, as well as geographic and historical information of the analyzed populations.

In conclusion, the results of this study demonstrate moderate level of genetic diversity and differentiation among populations, but low to moderate level of genetic diversity within populations. Preventing loss of further alleles with low genetic diversity in populations studied should be considered by implementing effective breeding strategies to reduce inbreeding and increase heterozygosity. Also, the two major phylogenetic clusters clearly showed the genetic relationship of the duck populations. Finally, we highlighted the usefulness of these microsatellite markers to evaluate genetic diversity and phylogenetic relationship in local duck populations of Indonesia. The results indicate the risk status and threats to the duck populations and are useful for designing conservation plans and developing future genetic improvement.

## Figures and Tables

**Table 1:** Genetic diversity analysis over all 22 loci and populations based on number of alleles (Na), observed (Ho) and expected heterozygosity (He), polymorphism information content (PIC), and *F*-statistics (*F*_IT_, *F*_IS_, and *F*_ST_)

No.	Locus	Chro. No./Linkage group	Na	N	Ho	He	PIC	*F*_IT_	*F*_IS_	*F*_ST_
1	AMU3	-	4	234	0.581	0.653	0.579	0.108	0.017	0.093
2	APH04	CAU6	7	234	0.256	0.553	0.475	0.538	0.427	0.195
3	APH20	8	4	234	0.274	0.590	0.509	0.536	0.470	0.125
4	APH24	CAU3	5	234	0.026	0.079	0.077	0.676	0.659	0.051
5	CAUD011	-	5	234	0.470	0.590	0.505	0.196	0.091	0.116
6	CAUD031	CAU1	7	234	0.427	0.479	0.449	0.102	0.043	0.061
7	CAUD035	CAU6	6	234	0.410	0.605	0.568	0.322	0.224	0.127
8	CAUD039	1	6	234	0.645	0.713	0.664	0.093	−0.001	0.093
9	CAUD111	5	6	234	0.333	0.633	0.592	0.472	0.389	0.135
10	CAUD128	CAU17	3	234	0.496	0.503	0.380	0.010	−0.068	0.072
11	CAUD040	CAU12	21	236	0.860	0.927	0.920	0.070	0.001	0.069
12	CAUD066	1	6	236	0.585	0.633	0.560	0.072	−0.035	0.103
13	AMU123	-	3	236	0.496	0.582	0.491	0.146	0.052	0.099
14	AMU52	10	6	236	0.254	0.551	0.492	0.536	−0.038	0.553
15	AMU68	CAU9	6	236	0.178	0.193	0.186	0.077	0.024	0.055
16	APH08	CAU6	8	236	0.436	0.743	0.700	0.412	0.024	0.398
17	CAUD005	CAU1	9	236	0.534	0.602	0.565	0.109	0.009	0.102
18	CAUD009	-	3	236	0.305	0.446	0.398	0.314	0.216	0.125
19	CAUD044	10	5	236	0.411	0.440	0.377	0.066	−0.060	0.119
20	CAUD086	CAU1	6	236	0.360	0.371	0.332	0.024	−0.099	0.112
21	CAUD132	27	5	236	0.466	0.646	0.569	0.278	0.157	0.143
22	CAUD048	11	22	238	0.866	0.916	0.908	0.053	−0.003	0.056
	Total		153							
	Average		6.955		0.440	0.566	0.513	0.237	0.114	0.137

N, number of individuals; *F*_IT_, global heterozygote deficit among eight duck populations; *F*_IS_, heterozygote deficit within duck populations; *F*_ST_, fixation index as genetic differentiation.

**Table 2:** Genetic diversity analysis within duck populations

Population	N	Na	Ho	He	*F*_IS_
AL	30	3.136	0.371	0.368	−0.011
BY	29	4.364	0.451	0.498	0.113
MG	30	4.682	0.450	0.545	0.152
MJ	28	3.818	0.464	0.486	0.037
PG	29	4.545	0.441	0.485	0.111
PT	30	4.227	0.396	0.443	0.084
RM	30	4.864	0.458	0.535	0.171
TR	30	4.864	0.488	0.519	0.077
Average	29.398	4.313	0.440	0.485	0.092

N, number of individuals analyzed; Na, number of alleles; Ho, observed heterozygosity; He, expected heterozygosity; *F*_IS_, heterozygote deficit within duck populations; AL, Alabio; BY, Bayang; MG, Magelang; MJ, Mojosari; PG, Pegagan; PT, Pitalah; RM, Rambon; TR, Turi.

**Table 3:** Pairwise population matrix of *F*_ST_ values and Nei’s genetic distances of eight duck populations

Population	AL	BY	MG	MJ	PG	PT	RM	TR
AL	0.000	0.295	0.133	0.196	0.270	0.298	0.271	0.335
BY	0.142	0.000	0.242	0.320	0.051	0.088	0.064	0.084
MG	0.076	0.086	0.000	0.093	0.262	0.290	0.176	0.210
MJ	0.099	0.120	0.038	0.000	0.307	0.367	0.226	0.274
PG	0.134	0.025	0.096	0.118	0.000	0.132	0.070	0.091
PT	0.155	0.045	0.109	0.142	0.067	0.000	0.116	0.133
RM	0.127	0.028	0.062	0.083	0.034	0.053	0.000	0.054
TR	0.149	0.033	0.073	0.100	0.040	0.061	0.021	0.000

Above diagonal and below diagonal, *F*_ST_ values and Nei’s genetic distances, respectively; AL, Alabio; BY, Bayang; MG, Magelang; MJ, Mojosari; PG, Pegagan; PT, Pitalah; RM, Rambon; TR, Turi.
